# Indirect comparison of efficacy between different antibiotic prophylaxis against the intracranial infection after craniotomy

**DOI:** 10.1186/s13756-020-00784-9

**Published:** 2020-07-31

**Authors:** Yulong Cao, Bin Wang, Jiao Shan, Zhizhong Gong, Jiqiu Kuang, Yan Gao

**Affiliations:** 1 Department of Hospital-Acquired Infection Control, Peking University People’s Hospital, No. 11 Xizhimen South Street, Xicheng District, Beijing, 100044 P. R. China; 2 Department of Neurosurgery, Peking University People’s Hospital, Beijing, 100044 P. R. China; 3 Department of Hospital-Acquired Infection Control, Beijing Jishuitan Hospital, Beijing, 100035 P. R. China; 4 School of Public Policy & Management of Tsinghua University, Beijing, 100084 P. R. China

**Keywords:** Craniotomy, Intracranial infection, Antibiotic prophylaxis, Indirect treatment comparison

## Abstract

**Background:**

Many studies had shown that prophylactic use of antibiotics could significantly reduce the intracranial infection (ICI) rate of craniotomy. However, there has been no comparison of these antibiotics.

**Methods:**

An electronic database search was performed, from inception to June 102,020. Randomized controlled trials (RCT) using different intravenous antibiotics (IVA) against the ICIs after craniotomy were considered. The primary outcome was the incidence rates of ICIs. An indirect treatment comparison (ITC) was conducted to compare the protective effect among the diverse antibiotic prophylaxis to prevent ICIs after craniotomy. Risk of potential bias was assessed.

**Results:**

A total of 3214 patients after craniotomy in 11 studies were included, 159 patients experienced postoperative ICI, including 33 patients in the antibacterial group and 126 in the control group. The calculate results of meta-analysis showed that except fusidic acid, preoperative intravenous injection of cephalosporin, clindamycin, vancomycin, and penicillin can significantly reduce the incidence of ICI after craniotomy, and ITC showed there was no statistically significance difference in the rates of post craniotomy ICI between the various antibiotics.

**Conclusion:**

The current evidence shows that low-grade antibacterial drugs can be selected to prevent ICI after craniotomy, but this may be due to the limited number of studies per antibiotic. It still needs more high-quality, large sample RCT to confirm.

**Systemic review registration:**

PROSPERO CRD42019133369.

## Introduction

Postoperative intracranial infections (ICIs) do not often occur but have potentially serious consequences [1]. One of the greatest risks for these infections is undergoing craniotomy. The rate of ICIs reached as high as 4.3 ~ 7.4% in some developing countries, although the aseptic technique was developed in recent years [2, 3], more accounted for cleaning incision surgical site infections [4]. ICIs can cause severe complications and poor outcomes, even death. In order to reduce the incidence of ICIs, preoperative prophylactic use of antibiotics is still an important principle for reducing ICIs.

Antibiotic prophylaxis is recommended in patients undergoing craniotomy. The conclusion of many surgical research also supported that all kinds of antibiotics can be used to prevent ICIs after craniotomy [5], and yet there are no original studies of direct comparison between these antibiotics.

When no head-to-head clinical trials comparing alternative treatments are available it is, considered appropriate to undertake an indirect treatment comparison (ITC). Therefore, randomized controlled trials of commonly used intravenous antibiotics (IVA) drugs for preventing ICIs were performed in an ITC to provide evidence for the prevention strategies of ICIs.

## Methods

This study was conducted following a protocol registered with PROSPERO (number CRD42019133369), and reported according to the Preferred Reporting Items for Systematic Reviews Incorporating Network Meta-analyses (PRISMA-NMA) guidelines [6]. A completed PRISMA- NMA recommendation checklist is revealed as an additional file (Additional file 1).

### Search strategy and eligibility criteria

The initial search in the Cochrane Library, PubMed, Embase, SinoMed, CNKI, VIP, and Wanfang database from inception to 10 June, 2020 included clinical studies that compared at least 2 interventions for the prevention of ICIs in patients after craniotomy. No language restriction was applied. RCTs were considered for this indirect comparison, irrespective of publication status. However, the literature on the following conditions will be excluded: 1) emergency brain injury, decompressive craniectomy, 2) preoperative prophylaxis is a combination of two or more antibiotics. Details of the search strategy are provided in Additional file 2.

### Study selection and data extraction

The full texts of every retrieved potentially relevant studies were obtained and two of the reviewers (CYL and WB) scrutinized these reports independently to determine which studies were required for further assessment. Differences in eligibility assessments were resolved by discussion and when necessary a final consensus was reached with the assistance of a third reviewer. Relevant data from each article were abstracted by 2 reviewers using a standardized extraction form. The extracted data included study characteristics, patient characteristics, interventions, outcomes, and relevant findings. Previous literature shows that few studies have met the criteria [7]. Therefore, there was no limitation on antibiotic classes, dose, full name, manufacturers and companies in this study.

### Quality control

The risk of bias for each selected studies were evaluated by Cochrane risk of bias tool, which included the following items: random sequence generation, allocation concealment, blinding of participants and personnel, blinding of outcome assessment, incomplete outcome data, selective reporting bias, and other biases [8]. After evaluating independently by two reviewers (CYL and WB), the assessment level of each papers were discussed by two or three reviewer if necessary. Each potential item was graded as high, low, or unclear level of bias.

### Statistical analysis

Two types of meta-analyses were performed. First, pairwise meta-analysis was used to assess the risk of ICIs after craniotomy with different IVA. The risk ratio (RR) and 95% confidence interval (CI) were calculated and forest plots were created using RevMan 5.3.3. Publication bias was examined by a comparison-adjusted funnel plot. Secondly, when no head-to-head RCT is available. The placebo then became a common comparator or a bridge between these antibiotics. We used ITC application for this analysis using Bucher method [9]. Random-effects modelling was selected a priori over fixed effects models taking into account both within and between studies heterogeneity given differences in RCT design. Therefore, the strength of randomization is partially maintained and any differences between treatments that are observed through an indirect comparison are less likely due to patient differences unrelated to the treatment [10]. Indirect comparisons and statistical tests were presented using STATA version 14.2 (Stata Corp., College Station, TX, USA) software.

## Results

### Literature identified

**Fig. 1:**
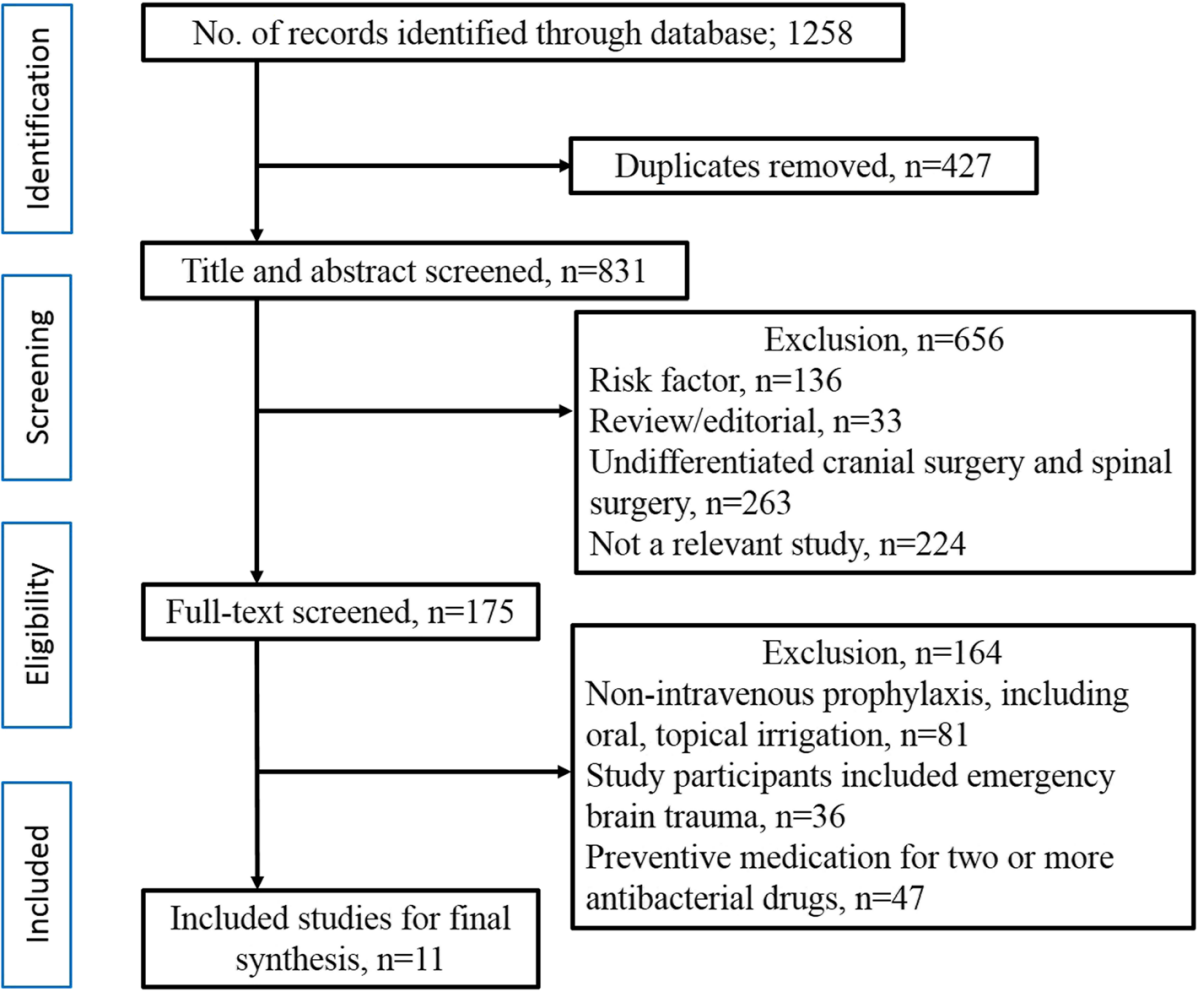
PRISMA flow diagram of identification and selection of studies

Of 1258 potentially relevant articles identified in the electronic database, 831 remained after the removal of duplicate records in Endnote X9. Overall, 175 potentially eligible articles were retrieved in full text. One hundred and sixty-four articles were excluded, not reporting the outcomes of interest, not providing a complete outcome data, comparing interventions in the same category. Finally, 11 trials studying 5 different antibiotics met the criteria and were included in the ITC. Eligible studies of comparison between intravenous antibiotics with ICIs are shown in Fig. 1.

### Evidence network and characteristics of the literature

**Fig. 2:**
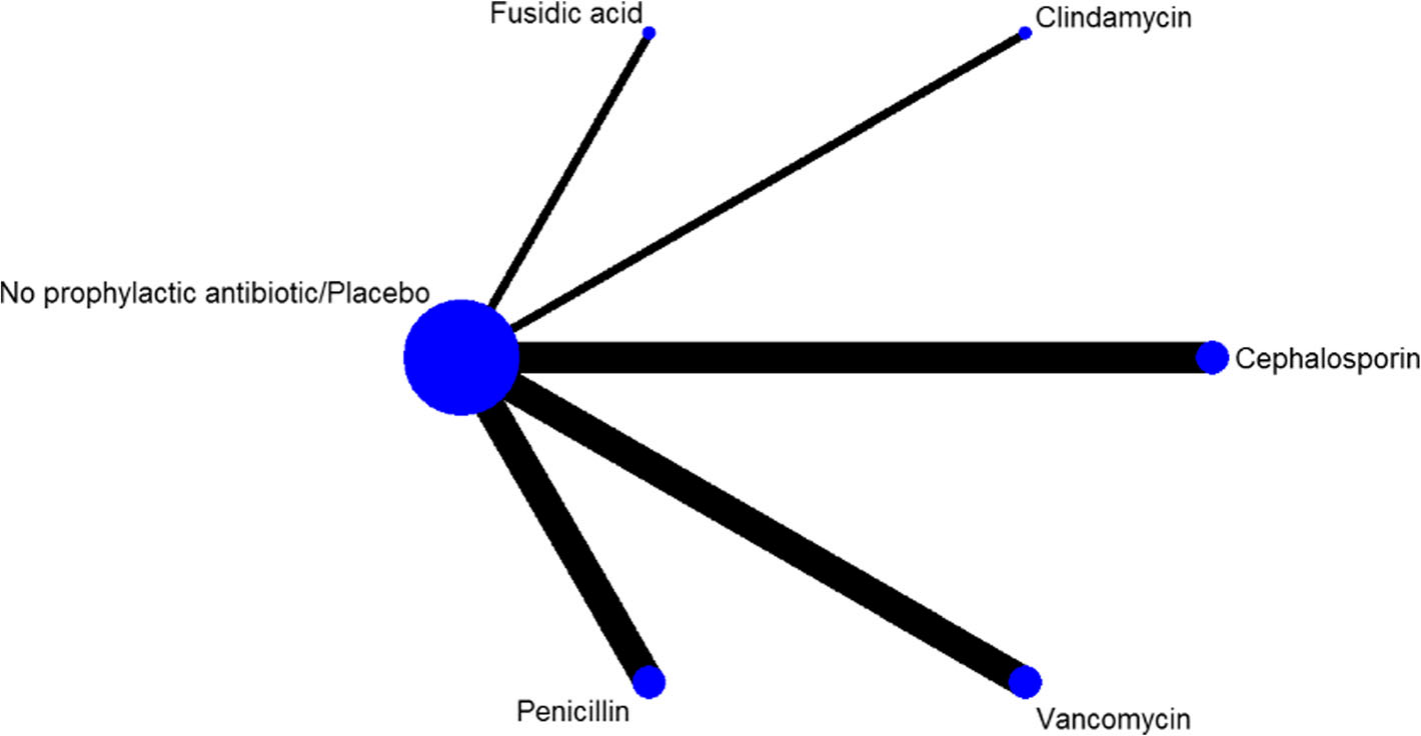
Network structure of intervention measures

Six different interventions were included in this study: cephalosporin, clindamycin, penicillin, vancomycin, fusidic acid, and no prophylactic postoperative antibiotic/placebo. The network relationship among the six intervention measures is shown in Fig. 2. Of the 11 studies, all the control groups were no prophylactic antibiotic/placebo; In treatment group, three articles using cephalosporin, three using vancomycin, three using penicillin, one using clindamycin, and one using fusidic acid. Details of the characteristics of the included studies are shown in Table 1.

**Table 1:** Characteristics of the included articles in the systematic review and meta-analysis

Autho (Year)	Country	Participants	Type of intervention (manufacturers/companies)	Treatment group ICIs rate	Type of control	Control group ICIs rate
Savitz and Malis.(1976) [11]	USA	Craniotomy	200 mg Clindamycin IV (N/A)	1/60	No prophylactic antibiotic	9/50
Geraghty et al.(1984) [12]	Ireland	Craniotomy+Burr hole	1 g Vancomycin IV (N/A)	0/72	No prophylactic antibiotic	5/67
Young et al.(1987) [13]	USA	Craniotomy+Stereotaxic procedures+Shunt placement	1 g Cefazolin IV (N/A)	3/286	No prophylactic antibiotic	13/301
Blomstedt et al.(1988) [14]	Finland	Supratentorial+suboccipital Craniotomy	1 g Vancomycin IV (N/A)	3/169	No prophylactic antibiotic	14/191
Bullock et al.(1988) [15]	South Africa	Craniotomy+VP shunt	2 g Piperacillin sodium IV (N/A)	2/141	Placebo	10/159
Van Ek et al.(1988) [16]	Netherlands	Craniotomy with Bone flap+subdual drainage+insertion+interval shunt+ommaya reseroir	1 g Cloxacillin IV (N/A)	6/183	Placebo	20/195
Blum et al.(1989) [17]	Germany	Shunt	50 mg/kg Cefazedone IV (Refosporin^R^, E. Merck)	3/50	No prophylactic antibiotic	7/50
Djindjian et al.(1990) [18]	France	Cerebral tumor+Meningioma+Vascular+Posterior fossa+stereotactic	1 g Oxacillin IV (N/A)	1/148	Placebo	7/153
Gaillard et al.(1991) [19]	Germany	Craniotomy	2 g Cefotiam IV (N/A)	12/356	Placebo	32/355
Mindermann et al.(1993) [20]	Switzerland	Craniotomy+Posterior fossa+Cranioplast+Ventricular-peritoneal shunting	500 mg Fusidic acid IV (Leo Pharmaceutical Products, Zuirich, Switzerland)	1/41	Placebo	4/44
Huang W. et al.(2009) [21]	China	Craniotomy	500 mg Vancomycin IV (Eli Lilly Japan K. K, Seishin Laboratories)	1/92	No prophylactic antibiotic	5/51

### Risk of bias

**Fig. 3:**
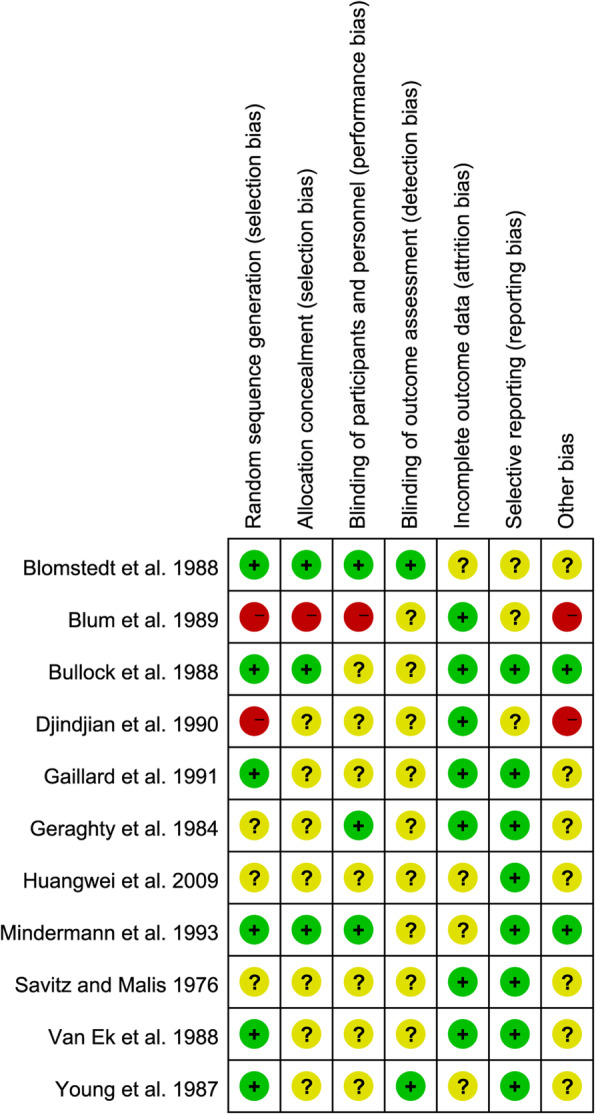
Risk of bias assessment for studies included in the indirect comparison

When evaluating the bias risk of the 11 studies, six studies have a low risk of bias for random sequence generation (selection bias), seven studies have an unclear risk of bias for allocation concealment, seven studies had an unclear risk of bias for blinding of participants and personnel (performance bias), only two had a low risk of bias for blinding of outcome assessment, seven studies had a low risk of bias for incomplete outcome data (attrition bias) and eight studies had a low risk of bias for selective reporting (reporting bias). The results of the risk of bias assessment are shown in Fig. 3.

### Effect of different IVA against ICIs

**Fig. 4:**
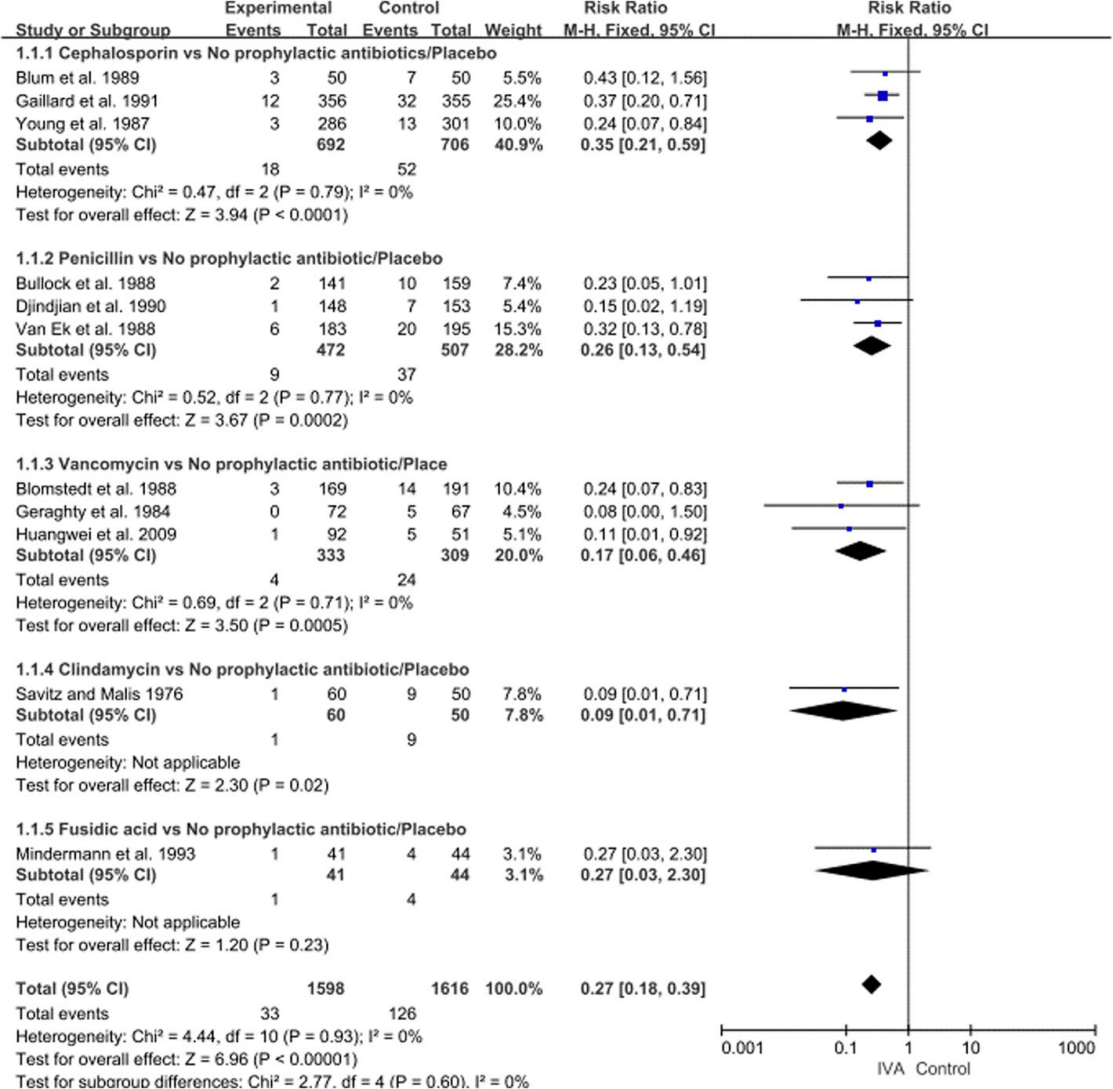
Forest plot dispalying fixed-effect meta-analysis of different IVA against ICIs. OR, odds radio; CI, confidence interval

The forest plots of risk differences (Fig. 4) showed that, no significant heterogeneity within pairwise comparisons of antibiotics was found except fusidic acid. The results of pairwise meta-analysis were as follows: Cephalosporin vs. No prophylactic antibiotic/Placebo, OR 0.35, 95% CI 0.21–0.59 (p < 0.01); Penicillin vs. No prophylactic antibiotic/Placebo, OR 0.26, 95% CI 0.13–0.54 (p < 0.01); Vancomycin vs. No prophylactic antibiotic/Placebo, OR 0.17, 95% CI 0.06–0.46 (*p* = < 0.01); Clindamycin vs. No prophylactic antibiotic/Placebo, OR 0.09, 95% CI 0.01–0.71 (*p* = 0.02); Fusidic acid vs. No prophylactic antibiotic/Placebo, OR 0.27, 95% CI 0.03–2.30 (*p* = 0.23).

In the direct comparison, cephalosporins, clindamycin, vancomycin, penicillin can significantly reduce the incidence of ICIs after craniotomy. However, there was no statistically significance difference between above four antibacterial drugs in the indirect comparison. (Table 2).

**Table 2:** Results of indirect comparison of 6 intervention methods

Cephalosporin	Penicillin	Vancomycin	Clindamycin	Fusidic acid	No prophylactic antibiotic/placebo
1.35 (0.56, 3.24)	–				
2.06 (0.66, 6.45)	1.53 (0.44, 5.30)	–			
3.89 (0.43, 34.85)	2.89 (0.31, 27.33)	1.89 (0.18, 20.05)	–		
1.30 (0.14, 12.06)	0.96 (0.10, 9.45)	0.63 (0.06, 6.92)	0.33 (0.02, 6.98)	–	
**0.35 (0.21, 0.59)**	**0.26 (0.13, 0.54)**	**0.17 (0.06, 0.46)**	**0.09 (0.01, 0.71)**	0.27 (0.03, 2.30)	–

**Fig. 5:**
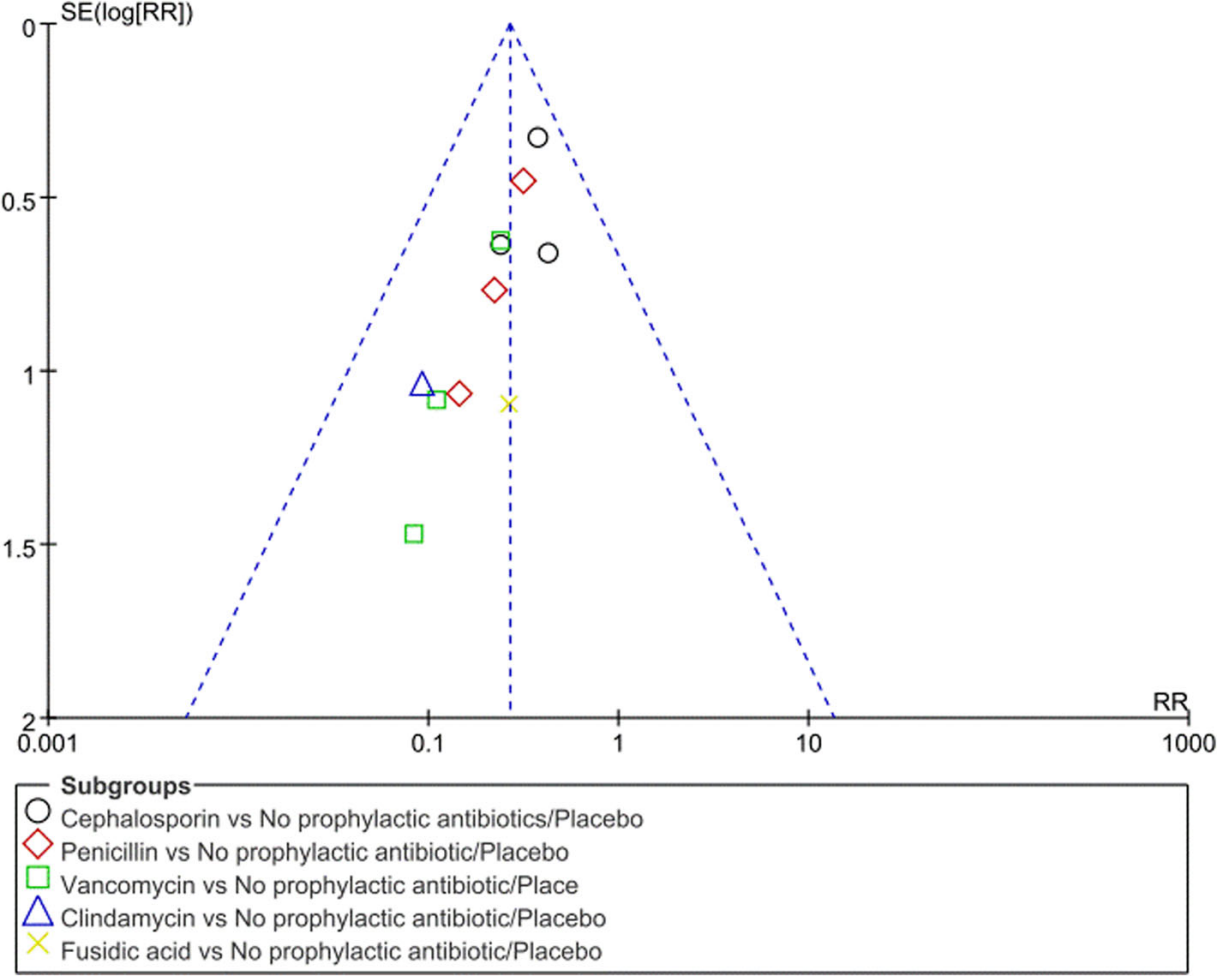
Funnel plot of the included studies

An analysis of publication bias on efficient indicators by funnel plot was shown in Fig. 5, the funnel plot showed no evidence of publication bias.

## Discussion

As early as the 1980s and 1990s, some scholars suggested that the preventive application of antibacterial drugs in elective surgery is not a protective factor, and the indiscriminate use will promote the emergence of multi-drug resistant bacteria [22]. Subsequently, some randomized controlled studies were published. Based on these data, some meta-analysis demonstrated that prophylactic antibiotics can significantly reduce the incidence of postoperative ICIs in the brain, 8.80% reduced to 1.90% [7, 23]. Therefore, prophylactic antibiotics have become the strategies adopted by most doctors to prevent postoperative infections.

How to provide targeted prevention measures for ICIs after craniotomy is increasingly valued by neurosurgical staff and infection preventionists. However, types of antibacterial drugs used and the mode of administration in the published researches were numerous [24], and the relative effects of the multiple antibacterial drugs were not analyzed.

To our knowledge, this study is the first ITC analysis provides the most recent and comprehensive analysis of the effectiveness of different antibiotic prophylaxis for the prevention of ICIs after craniotomy. More than 3000 patients were enrolled from 11 studies. Analogous to previous meta-analyses, our findings indicate that prophylactic use of intravenous antibiotics to decrease the incidence of ICIs after craniotomy. The results showed that, except fusidic acid, preoperative intravenous injection of cephalosporin, clindamycin, vancomycin, and penicillin can significantly reduce the incidence of ICIs after craniotomy, there was no statistically significance difference between four antibiotics in the indirect comparison.

Oral fusidic acid is an antibacterial agent used for the treatment of staphylococci infections in the skin and soft tissue. In step 1, pairwise meta-analysis, findings found that intravenous fusidic acid was associated with increased odds of surgical site infections compared with other antibiotics. In addition, some studies suggest that not through intravenous injection fusidic acid, in order to avoid thrombophlebitis even subcutaneous tissue necrosis, may also lead to reversible jaundice [25]. Based on this, we do not recommend intravenous fusidic acid for prophylactic use.

By ITC, with regard to efficacy, clindamycin ranked first. After clindamycin enters the patient through an intravenous drip, the plasma concentration is quite high, and it is prevalent among the patient. It can reach an effective antibacterial level in most tissues and body fluids in a short time [26]. Not only can it produce relatively strong antibacterial activity against common gram-positive bacteria [27], but it can also be effective against anaerobic bacteria, so it effectively reduces the number of intracranial infection after craniotomy. And the drug is mainly metabolism by the liver of the human body after administration, and excreted through the bile and feces, and a part can be excreted through the urine. After the drug is utilized, it will not result in adverse effects on the liver and kidney function of the patient, so clindamycin is safe. In addition, clindamycin is cheap, cost-effective in pharmacoeconomic evaluation [28]. Considering its safety, good pharmacokinetics and acceptable price, clindamycin has been the first choice for prophylactic antibiotics for preoperative brain surgery.

Cephalosporins include: cefazolin, cefazedone and cefotiam, both first and second generation cephalosporins, mainly used to treat skin infections caused by Gram-positive cocci and streptococci. These bacteria are considered to be the main pathogens causing SSI due to improper skin disinfection in the preoperative surgical site [29]. Cephalosporins can be used as pre-operative preventive medications for most cleansing or cleaning-contamination procedures, but not as the first choice for intravenous prophylaxis.

With the emergence of MRSA, the use of vancomycin has increased rapidly. Although some people think that prophylactic use of vancomycin is more broad-spectrum antibiotics or combined with multiple antibiotics will reduce the production of drug-resistant bacteria, but with the widespread use of vancomycin, the infection rate of vancomycin-resistant enterococci is also increasing [30]. Since widespread use of vancomycin increases the chance of vancomycin-resistant cocci infection, prophylactic vancomycin is now only used in patients who are allergic to penicillin or cephalosporin or have a history of MRSA infection [31]. Therefore, this study recommends a single IVA for elective type I clean surgical incision craniotomy, in principle no more than 24 h, with risk factors can be extended to 48 h, should use broad-spectrum anti-emergence, can pass the normal blood-brain barrier, necessary When referring to the in-hospital strain spectrum, select more sensitive drugs.

The results of this ITC should be interpreted with the consideration of several limitations. First, due to the limited data, inconsistency analysis could not be conducted. Nevertheless, the heterogeneity of the included studies was determined through pairwise and sensitivity analyses to comply with the assumption of indirect comparison. In addition, we included five intravenous interventions and no prophylactic antibiotic (placebo), the number of studies included in the analysis was relatively small, we could not make head-to-head comparisons in this study based on the limited sample size. Evidence is scant, mostly indirect and do not have any direct comparisons between different antibiotics. We found that clindamycin is the most effective antibiotic against ICIs after craniotomy based on the ITC, these indirectly driven comparison on the strength of direct evidence provided by clinical trials, ranking of drug effectiveness are making inferences about a causal claim. This makes it necessary to consider the actual situation of patients when using the results of this study as an evidence. Second, our meta-analysis relied on study-level and not patient-level data. From the perspective of the quality of the included studies, although there are six studies that refer to the random allocation method using the random number table method; However, many studies do not mention the blinding of participants and personnel (performance bias) and blinding of outcome assessment (detection bias), and no intentional analysis is performed, thus affecting the results and its conclusion strength. Owing to these problems, the extrapolation of the results of this study is restricted to a certain extent.

Research on the selection of preventive antibiotics for craniotomy will continue, but it has been acknowledged that the widespread use of antibacterials can cause severe drug resistance and begin to develop strict antibiotic use strategies. This study might provide new insights of prophylaxis choices against the ICIs after craniotomy whilst awaiting the arrival of higher quality evidence. Large-scale, multi-center, high-level research evidence is urgently needed to guide the application of prophylactic antibiotics for craniotomy to ensure the clinical safety of patients and improve the severe bacterial resistance.

## Conclusion

The use of prophylactic antibiotics significantly decreases the rate of ICIs craniotomy. The current evidence shows that there was no statistically significance difference between the different antibiotics, low-grade antibiotics can be selected to prevent ICIs after craniotomy. However, it still need more high-quality, large sample RCT to confirm.

## Supplementary information

**Additional file 1.** PRISMA NMA Checklist of Items to Include When Reporting A Systematic Review Involving a Network Meta-analysis

**Additional file 2.** Serch strategy

## Data Availability

All data are fully available without restriction.
