# Supplementary material for: Indirect comparison of efficacy between different antibiotic prophylaxis against the intracranial infection after craniotomy
Source: Antimicrob Resist Infect Control. 2020 Jul 31;9:122. doi: 10.1186/s13756-020-00784-9 (PMC7393706; doi:10.1186/s13756-020-00784-9)
Supplement: Supplementary file 2 — Additional file 2. Serch strategy [file 13756_2020_784_MOESM2_ESM.docx]

**Supplemental file 2:** **Serch strategy**

**PubMed**

("Craniotomy"[Mesh] OR "Neurosurgical Procedures"[Mesh:NoExp] OR

"Neurosurgery"[Mesh] OR "Intracranial Pressure"[Mesh] OR "Brain/surgery"[Mesh] OR craniotom*[tw] OR neurosurg*[tw] OR brain surg*[tw] OR intracranial pressure[tw])

AND

("Antibiotic Prophylaxis"[Mesh] OR "Cephalosporins"[Mesh] OR "Penicillins"[Mesh] OR "Ampicillin"[Mesh] OR "Carbapenems"[Mesh] OR "Vancomycin"[Mesh] OR "Gentamicins"[Mesh] OR "Clindamycin"[Mesh] OR cephalosporin*[tw] OR penicillin*[tw] OR vancomycin*[tw] OR Cefazolin[tw] OR Cefotaxime[tw] OR Cefixime[tw] OR Cefmenoxime[tw] OR Cefotiam[tw] OR Ceftizoxime[tw] OR Ceftriaxone[tw] OR Cefuroxime[tw] OR Cephalexin[tw] OR Cefaclor[tw] OR Cefadroxil[tw] OR Cefatrizine[tw] OR Cephaloglycin[tw] OR Cephradine[tw] OR Amdinocillin[tw] OR Amdinocillin Pivoxil[tw] OR Cyclacillin[tw] OR Methicillin[tw] OR Nafcillin[tw] OR Oxacillin[tw] OR Cloxacillin[tw] OR Dicloxacillin[tw] OR Floxacillin[tw] OR Penicillanic Acid[tw] OR Penicillin G[tw] OR Ampicillin[tw] OR Amoxicillin[tw] OR Carbenicillin[tw] OR Penicillin G Benzathine[tw] OR Penicillin G Procaine[tw] OR Sulbenicillin[tw] OR Penicillin V[tw] OR Sulbactam[tw] OR Ticarcillin[tw] OR Azlocillin [tw] OR Mezlocillin [tw] OR Piperacillin [tw] OR Pivampicillin[tw] OR Talampicillin [tw] OR meropenem[tw] OR Gentamicins[tw] OR Gentamycin[tw] OR Clindamicin[tw] OR Clindamycin [tw] )

AND

("postoperative infection"[Mesh] OR "Surgical Wound Infection"[Mesh] OR Postoperative Wound Infection[All Fields] OR Surgical Wound Infections[All Fields] OR Surgical Site Infections[All Fields])

AND

("randomized controlled trial" [pt] OR "controlled clinical trial" [pt] OR "randomized" [tiab] OR "placebo" [tiab] OR "clinical trials as topic" [mesh: noexp] OR "randomly" [tiab] OR "trial" [ti]) NOT ("animals" [mh] NOT "humans" [mh])

**EMBASE**

(exp craniotomy/ or craniotomy.tw. or exp neurosurgery/ or neurosurgery.tw. or exp intracranial pressure/)

and

(exp postoperative infection/ or exp intracranial infection.tw.)

and

(exp antibiotic prophylaxis/ or exp cephalosporin derivative/

or exp penicillin derivative/

or exp ampicillin/

or exp carbapenem derivative/

or exp vancomycin/

or exp gentamicin/

or exp clindamycin/

**Cochrane library**

craniotom* OR neurosurg*

AND

"surgical infection*" OR "wound infection*" OR intracranial infection*"

AND

"antibiotic prophylaxis" OR Cephalosporin* OR Penicillin* OR vancomycin* OR Cefazolin OR

Cefotaxime OR Cephalexin OR gentamycin
